# Coinfection with *Tritrichomonas foetus* and *Giardia duodenalis* in Two Cats with Chronic Diarrhea

**DOI:** 10.1155/2016/5705168

**Published:** 2016-09-06

**Authors:** Sergio A. Zanzani, Alessia L. Gazzonis, Paola Scarpa, Emanuela Olivieri, Hans-Jörg Balzer, Maria Teresa Manfredi

**Affiliations:** ^1^Department of Veterinary Medicine, Università degli Studi di Milano, Milano, Italy; ^2^Department of Veterinary Medicine, Università degli Studi di Perugia, Perugia, Italy; ^3^IDEXX Laboratories, Ludwigsburg, Germany

## Abstract

*A Tritrichomonas foetus* and* Giardia duodenalis* mixed infection was diagnosed in two Maine Coon cats aged six months. One of them presented a history of chronic liquid diarrhea and of several unsuccessful treatments. In both cats,* G. duodenalis* and trichomonads were detected in fecal smears from freshly voided feces; the presence of* T. foetus* was confirmed by a real-time PCR assay. The cats completely recovered after treatment with ronidazole. In a refrigerated fecal sample collected from the cat with chronic diarrhea, drop-shaped trichomonad pseudocysts smaller than* G. duodenalis* cysts were detected. They appeared brownish or light-bluish when stained with Lugol's solution or with Giemsa stain, respectively, and their morphological features were similar to those expressed by bovine* T. foetus* pseudocysts* in vitro*. Existence of pseudocysts even in feline trichomonads is noteworthy as they could represent a form of protozoan resistance due to unfavorable conditions whose detection in refrigerated feces can be a useful clue for clinicians.

## 1. Introduction

*Tritrichomonas foetus*, agent of bovine trichomonosis, was recently recognized as a primary cause of feline trichomoniasis, a large bowel disease characterized by intermittent or chronic diarrhea mainly occurring in multihoused cats from catteries or shelters [1–3]. The infection was frequently diagnosed in cats younger than 1 year with worldwide distribution [4]. Similar to other trichomonads, for example, those infecting humans,* T. foetus* presents only a trophozoite stage although a pseudocyst stage was described for the bovine isolate [5, 6].* Giardia duodenalis* is an intestinal protozoan with a large diffusion and prevalence values highly variable in domestic cats [7–9]. Several surveys showed that cats host specific or zoonotic* Giardia* assemblages [7, 9, 10].* Giardia* has often been found in the feces of diarrheic cats singly or in coinfection with* T. foetus* [11]. However, reports of coinfection with both of these enteropathogens are limited, and no pseudocyst stage of* T. foetus* in cat feces was previously reported [5]. Further, ronidazole was documented to be effective for the control of* Tritrichomonas* infection in cats whereas its efficacy against* Giardia* was demonstrated only in dogs [12]. This article reports a coinfection with* T. foetus* and* G. duodenalis* in two owned cats and the pseudocyst stage of* T. foetus* in feline feces with its morphology.

## 2. Case Presentation

Two littermate Maine Coon females aged six months underwent examination by the referring veterinarian as one of them presented a 3-month history of liquid malodorous diarrhea. A previous diagnosis following coprological analyses in both cats had indicated an infection sustained by ascarids and the animals had been treated by practitioners with milbemycine oxime and praziquantel (2 mg/kg bw and 5 mg/kg bw, resp., PO, single administration). Due to persistent diarrhea, in the affected cat coprological analyses were repeated to verify both effectiveness of treatment against ascarids and a possible infection with* Giardia*. The cat resulted in being positive for* Giardia* coproantigens (IDEXX SNAP®* Giardia* Test, IDEXX Laboratories, Hoofddorp, Netherlands) and was treated with fenbendazole (50 mg/kg bw, PO, SID) for 5 days, obtaining only a moderate and transient improvement of feces consistence. As some weeks after this treatment liquid diarrhea continued, another fecal test was performed revealing the persistence of* Giardia* coproantigens. A treatment with spiramycin and metronidazole (75000 IU/kg bw 12.5 mg/kg bw, PO, SID) followed for 10 days. The feces became formed and no longer malodorous, but few days after treatment signs recurred. In the meantime, the two cats still continued to use the same litter and even the one whose feces had always been formed began to present mucous diarrhea. Thus, fecal samples collected from the two animals were submitted to the Veterinary Parasitology Laboratory of University of Milan for parasitological evaluation. Overall, parasitological analysis was performed on two fecal samples for each cat. The first two samples were analyzed after refrigeration in the same day, whereas the following samples were analyzed fresh having them delivered within almost 30 minutes after defecation. Centrifugation-flotation technique by NaNO_3_ solution (s.g. 1200 g/L), fresh fecal smears stained with Lugol's solution, and* Giardia* and* Cryptosporidium* coproantigens detection by an available commercial kit (RIDA®QUICK* Cryptosporidium*/*Giardia* Combi, R-Biopharm AG, Darmstadt, Germany) were performed.

**Figure 1:**
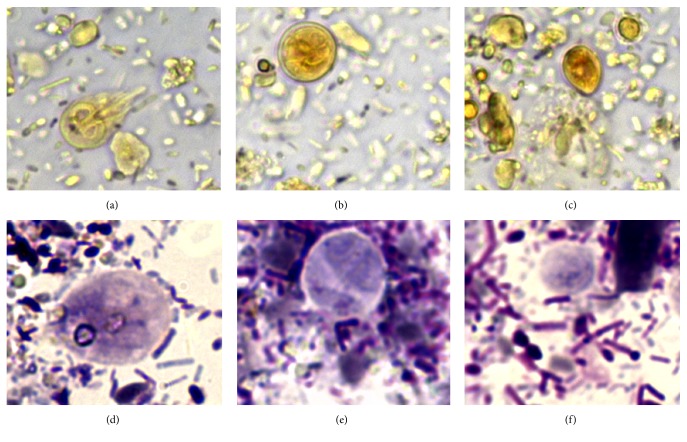
Fecal smears from a 6-month-old female Maine Coon cat with chronic liquid diarrhea stained with Lugol's solution (a–c) and Giemsa stain (d–f); (a) and (d) showed* Giardia duodenalis* trophozoite; (b) and (e) showed a* Giardia duodenalis* cyst; (c) and (f) showed drop-shaped trichomonads (630x).

**Figure 2:**
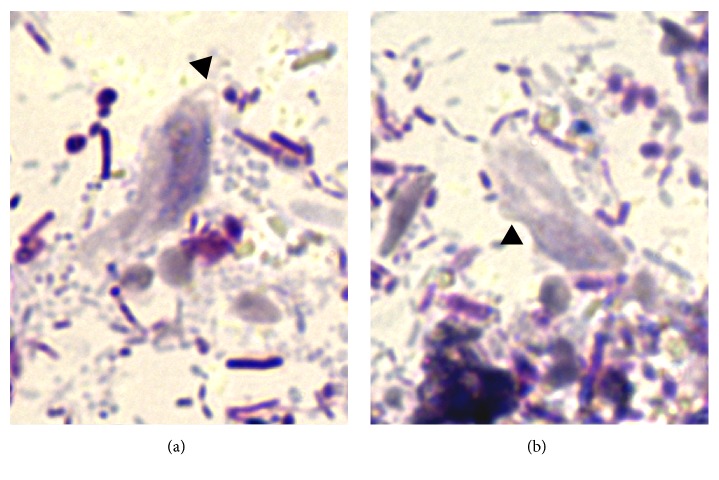
Trichomonads in fecal smear from the cat with diarrhea. Arrow heads in (a) indicate anterior flagella emerging from the trophozoite, while arrow heads in (b) indicate undulating membrane (1000x).

**Figure 3:**
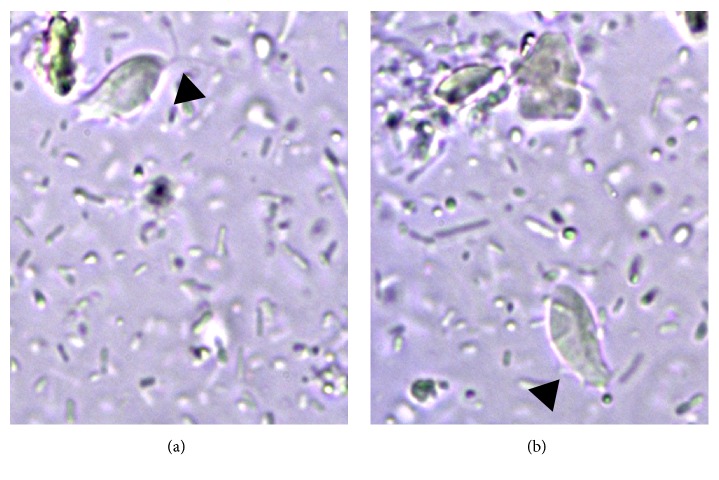
Typical morphology of trichomonads observed in saline solution-diluted fresh fecal smear from the cat with diarrhea. Arrow heads in (a) and in (b) indicate anterior flagella and undulating membrane, respectively (630x).

**Figure 4:**
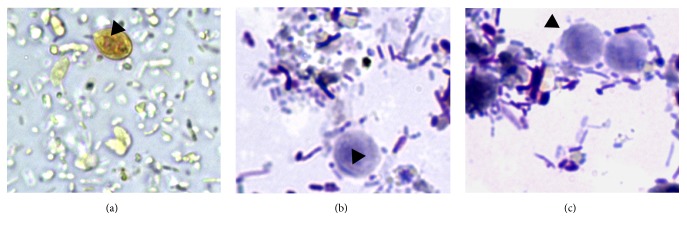
Drop-shaped unidentified elements in fecal smears stained with Lugol's solution (a) and Giemsa stain (b-c). Arrow heads in (a) indicate an internal oval structure (400x). Arrow heads in (b) indicate a curved linear structure (1000x). Arrow heads in (c) indicate an undulated portion of the surface (1000x).

No protozoan cysts or trophozoites and no ova of helminths were detected by centrifugation-flotation technique in both cats, whereas they were positives to* Giardia*-coproantigens. However, in the fecal smear stained with Lugol's solution obtained from the refrigerated sample of the cat with chronic liquid diarrhea, several cysts and trophozoites of* G. duodenalis* and unidentified elements were found. The latter appeared smaller (length: average 8.18 *μ*m, min–max 6.98–8.88 *μ*m; width: average 6.35 *μ*m, min–max 6.06–6.83 *μ*m) than cysts and trophozoites of* G. duodenalis*, were drop-shaped and brownish in color. An additional Giemsa stained fecal smear confirmed the presence of the unidentified drop-shaped elements (DSE) together with* G. duodenalis* cysts and trophozoites (Figure 1) and detected other elements showing clear morphological features of trichomonads trophozoites (Figure 2). At Giemsa staining, DSE appeared stained light-bluish; they presented a partially smooth surface, an undulated portion, and an internal curved linear structure, pink-violet stained, resembling the curved costa observed in bovine* T. foetus* living pseudocysts. In addition, some of DSE in the fecal smear stained with Lugol's solution presented an internal oval structure (Figure 3) [6, 13]. According to the morphological features of the parasitic elements, an infection sustained by* T. foetus* or by* Pentatrichomonas hominis* was then hypothesized. Analysis of the second fecal samples by saline solution-diluted fresh fecal smear confirmed only presence of trophozoites belonging to* T. foetus*/*P. hominis* showing an undulating membrane, the flagella, and a rapid forward motion (Figure 4). The fecal samples were processed for molecular analysis by a real-time PCR targeting* T. foetus* 5.8S rRNA gene (AF339736) that was performed at IDEXX Laboratories, Vet Med Labor GmbH, as previously described [11]. Molecular analyses of extracted nucleic acid from the fecal samples of the two cats confirmed the organism to be* T. foetus*. The diagnosis was mixed intestinal infection with* T. foetus* and* G. duodenalis* in both cats. Following the results of the latest parasitological analysis and, primarily, the diagnosis of* T. foetus* infection, the two animals were treated with ronidazole (30 mg/kg bw, PO, SID) for 14 days. Before suspending therapy, parasitological analyses and PCR assays were performed and the fecal samples tested negative. Six weeks after treatment, the owner reported that the two cats had formed feces and they still tested negative for parasitological analysis.

## 3. Discussion

*T. foetus* and* G. duodenalis* are both causative agents of diarrhea in cats, and their observed prevalence is extremely variable in owned cats. In a recent study, 0.7% of cats presented to a cat clinic were shedding* T. foetus*, whereas it in cat shows the prevalence of* T. foetus* infection exceeded 30% [2]. Prevalence of* G. duodenalis* in owned cats, estimated using detection of coproantigens, showed a high variability [7, 9, 14].* T. foetus* and* G. duodenalis* mixed infection in purebred cat is likely quite a common condition (prevalence = 4.35% to 22.72%) [15, 16]. Diagnosis of* T. foetus* from fecal samples can be performed by different methods such as copromicroscopic examination, fecal cultures (InPouch TF-Feline), and PCR. Cultures and PCR have been considered methods with high sensitivity; however, cultures need a long time of incubation (12 days) before a sample can be considered negative or positive for* T. foetus*. Further, in microscopic analysis of fecal smears or cultures aimed at searching for* T. foetus* trophozoites, the examined fecal samples should be from fresh voided feces or rectal swabs in which live trophozoites are more easily recognized. More recently, low specificity of cultures was demonstrated and a possible misdiagnosis of tritrichomonosis in cats using InPouchTM TF-Feline medium might occur [17]. In cats,* T. foetus* is considered trichomonads with no existing cyst form; nevertheless, formation of pseudocysts or of true cysts has been already observed in several trichomonads, probably as a response to environmental stress [18]. Pseudocysts with internalization of flagella were usually observed in* T. foetus* isolated from cattle both* in vitro* and* in vivo *[13, 18].* In vitro*, a large number of pseudocysts of bovine* T. foetus* can be obtained from cultures grown at 37°C when cooled to 4°C for 4 h [5]. DSE isolated in the feces of diarrheic cats had morphological features similar to those observed in bovine* T. foetus* living pseudocysts obtained* in vitro*. Particularly, they presented an internal oval structure resembling the nucleus and some undulations due to the movement of the internalized recurrent flagellum inside the cells of bovine* T. foetus* living pseudocysts recorded by differential interference contrast microscopy [5]. Even though further investigations should be performed under experimental conditions, DSE found in feline feces could be reasonably considered* T. foetus* pseudocysts. Moreover, their detection in fecal smears stained with Lugol's solution or Giemsa stain obtained from a refrigerated sample could be of particular interest for clinicians as positive control with supporting the diagnosis of* T. foetus* infections in cats and catteries [11, 19]. To date, the presence of trichomonads can be detected via light microscopy only in freshly voided feces [4]. Moreover, pseudocysts could represent a form of parasite resistance developing under unfavorable conditions, explaining both the observed environmental resilience of feline* T. foetus* in feces at room temperature and at +4°C after 24 h storage and their diffusion among feline hosts in shelters or catteries [20]. As for* Giardia*, the infection is usually treated with fenbendazole and metronidazole, whereas ronidazole is currently the treatment of choice against* T. foetus* [21]. In this case report, ronidazole was effective against both* T. foetus* and* G. duodenalis*. In addition, this is the first report showing the effectiveness of ronidazole against* G. duodenalis* in cats, as this medication had been previously successfully used against this agent only in kennel dogs [12].
